# Percutaneous Endoscopic Gastrostomy (PEG) Tube Migration Complicated With Acute Pancreatitis: A Clinical Case Study

**DOI:** 10.7759/cureus.72637

**Published:** 2024-10-29

**Authors:** Hezborn M Magacha, McKenna A Andrews, Sagar Nagpal, Venkata Vedantam

**Affiliations:** 1 Internal Medicine, East Tennessee State University Quillen College of Medicine, Johnson City, USA

**Keywords:** abdominal imagimg, elevated lipase, epigastric tenderness, pancreas disease, percutaneous endoscopic gastrostomy (peg) feeding

## Abstract

Acute pancreatitis is characterized by the premature activation of pancreatic enzymes leading to autodigestion and inflammation, commonly caused by gallstones or chronic alcohol consumption. However, iatrogenic factors, such as migration of a percutaneous endoscopic gastrostomy (PEG) tube, can be a cause of acute pancreatitis but are less frequently reported in the literature. This is a case of a 77-year-old male patient with a medical history significant for myasthenia gravis with dysphagia requiring a PEG tube placement who presented with abdominal pain and elevated lipase levels. Imaging revealed that the PEG tube had moved further inside the stomach compressing the pancreatic duct, causing acute pancreatitis. Repositioning the tube relieved the obstruction and normalized the patient's lipase levels, emphasizing the need for regular monitoring of PEG tube placement to prevent such complications. Clinicians should consider PEG tube migration in the differential diagnosis of acute pancreatitis to ensure timely and effective management.

## Introduction

Acute pancreatitis is an inflammatory condition of the pancreas that can result from a variety of etiologies, including gallstones, alcohol abuse, and iatrogenic causes, and symptoms range from mild abdominal symptoms to life-threatening complications with high mortality and morbidity [1]. The pathophysiology of acute pancreatitis involves the premature activation of the gland's pancreatic enzymes, leading to autodigestion and subsequent inflammatory response. Chronic pancreatitis involves repeated acute attacks that lead to inflammatory infiltrations and fibrosis within the pancreas [2]. 

The most common causes of acute pancreatitis include gallstones and chronic alcohol consumption. Gallstones, which can obstruct the pancreatic duct, account for approximately 40-70% of cases, making them the leading cause of acute pancreatitis. Chronic alcohol abuse, responsible for 25-35% of cases, induces acute pancreatitis by increasing the permeability of pancreatic ductal cells and promoting enzyme activation. Other etiologies include hypertriglyceridemia, hypercalcemia, certain medications, and genetic predispositions, each contributing to the pathogenesis through different mechanisms [3,4]. 

Acute pancreatitis is a rare complication of the percutaneous endoscopic gastrostomy (PEG) tube. Cases of acute pancreatitis secondary to PEG tube migration are relatively rare, but they have been documented in medical literature. A PEG tube is a medical device used for long-term enteral feeding in patients who cannot consume food orally. It is inserted through the abdominal wall into the stomach using endoscopic guidance. The PEG tube provides a way to deliver nutrition, fluids, and medications directly into the stomach, bypassing the oral route [4]. 

In this article, we present a case of a 77-year-old male patient who presented to the hospital with a complaint of abdominal pain and was diagnosed with acute pancreatitis secondary to PEG tube migration, four months later after having the PEG tube placed due to dysphagia secondary to myasthenia gravis.

## Case presentation

A 77-year-old male with a medical history significant for myasthenia gravis with oropharyngeal dysphagia status post PEG tube placement four months before this presentation, hypertension on losartan and amlodipine, and complete heart block status post cardiac pacemaker presented to the emergency department (ED) with a complaint of abdominal pain localized in the epigastric area. The patient reported that the pain had started the day before coming to the hospital. He noted that the pain was initially centered around the PEG tube site and subsequently intensified and became more pronounced in the epigastric area radiating to the back. The patient reported pain of 8/10 on a scale of 1-10. The patient also reported several episodes of nausea but no vomiting. He denied chills, fever, or diarrhea. The patient reported that his PEG tube seemed to have retracted and advanced further into his abdomen than usual. The patient denied any recent history of traumatic events or any newly started medications that could explain the current presentation.

Vitals on arrival to the ED were a temperature of 98°F, a pulse of 94 beats/min, respirations of 17, and a blood pressure of 143/82mmHg. Physical examination revealed that the patient was not in acute distress. Abdominal examination showed only mild tenderness to palpation, with no signs of peritoneal irritation. The PEG tube appeared to be correctly positioned; however, the hub was not secure and retracted back into the skin when pulled. 

Initial laboratory tests indicated mild hypokalemia and a significantly elevated lipase level of 1700 U/L, alongside an elevated white blood cell count (labs as shown in Tables 1-3). 

**Table 1:** Complete metabolic panel

Test	Result/Status	Units	Ref Range
Lipase	1761	U/L	8-78
EGFR-CKD-EPI	88	mL/min/1.7	
Sodium	142	mEq/L	137-145
Potassium	3.3	mEq/L	3.6-5.2
Chloride	108	mEq/L	98-107
CO2	28	mEq/L	22-29
Glucose	140	mg/dL	70-99
Urea nitrogen	25	mg/dL	5.0-25.0
Creatinine	0.9	mg/dL	07-1.3
Protein total	5.9	g/dL	6.3-8.2
Albumin	3.6	g/dL	3.5-5.0
Calcium	9.2	mg/dL	8.4-10.4
Total bilirubin	0.8	mg/dL	0.2-1.3
Alkaline phosphatase	48	U/L	40-150
ALT	8	U/L	0-55
AST	10	U/L	5-34

**Table 2:** Complete blood count

Test	Result/Status	Units	Ref Range
WBC	14.0	10(3)/mcL	4.8-10.5
RBC	4.2	10(6)/mcL	4.4-5.6
HGB	11.9	g/dL	13.6-17.3
HCT	37.1	%	39.5-51.7
MCV	88.1	fL	83.5-96.8
MCH	28.3	pg	27.3-33.3
MCHC	32.1	g/dL	32.9-34.6
RDW-CV	13.1	%	11.6-14.1
PLT	167	10(3)/mcL	166-383
MPV	8.8	fL	6.5-10.5
Neutrophils	82	%	44-72
Lymphocytes	10.7	%	20-49
Monocytes	6.0	%	4.5-13
Eosinophils	0.6	%	0-6
Basophils	0.4	%	0-2
Neutrophils absolute	11.5	10(3)/mcL	1.5-6.5
Lymphocytes absolute	1.5	10(3)/mcL	0.8-3.5
Monocytes absolute	0.8	10(3)/mcL	0.2-1.0
Eosinophils absolute	0.1	10(3)/mcL	0-0.4
Basophils absolute	0.1	10(3)/mcL	0-0.2
Immature granulocyte	0.3	%	0-2
Immature granulocytes abs	0.0	10(3)/mcL	0-7
Nucleated RBC/100WBC	0.0	#/100WBC	Ref:0

**Table 3:** Lipid panel

Component	Value	Reference	Unit
Cholesterol	128	0-200	mg/dL
LDL Chol	97	0-100	mg/dL
HDL	41	30-75	mg/dL
VLDL	16	5-40	mg/dL
Triglycerides	137	0-150	mg/dL
Chol/HDL ratio	3.12	0.0-5.0	mg/dL

**Figure 1:**
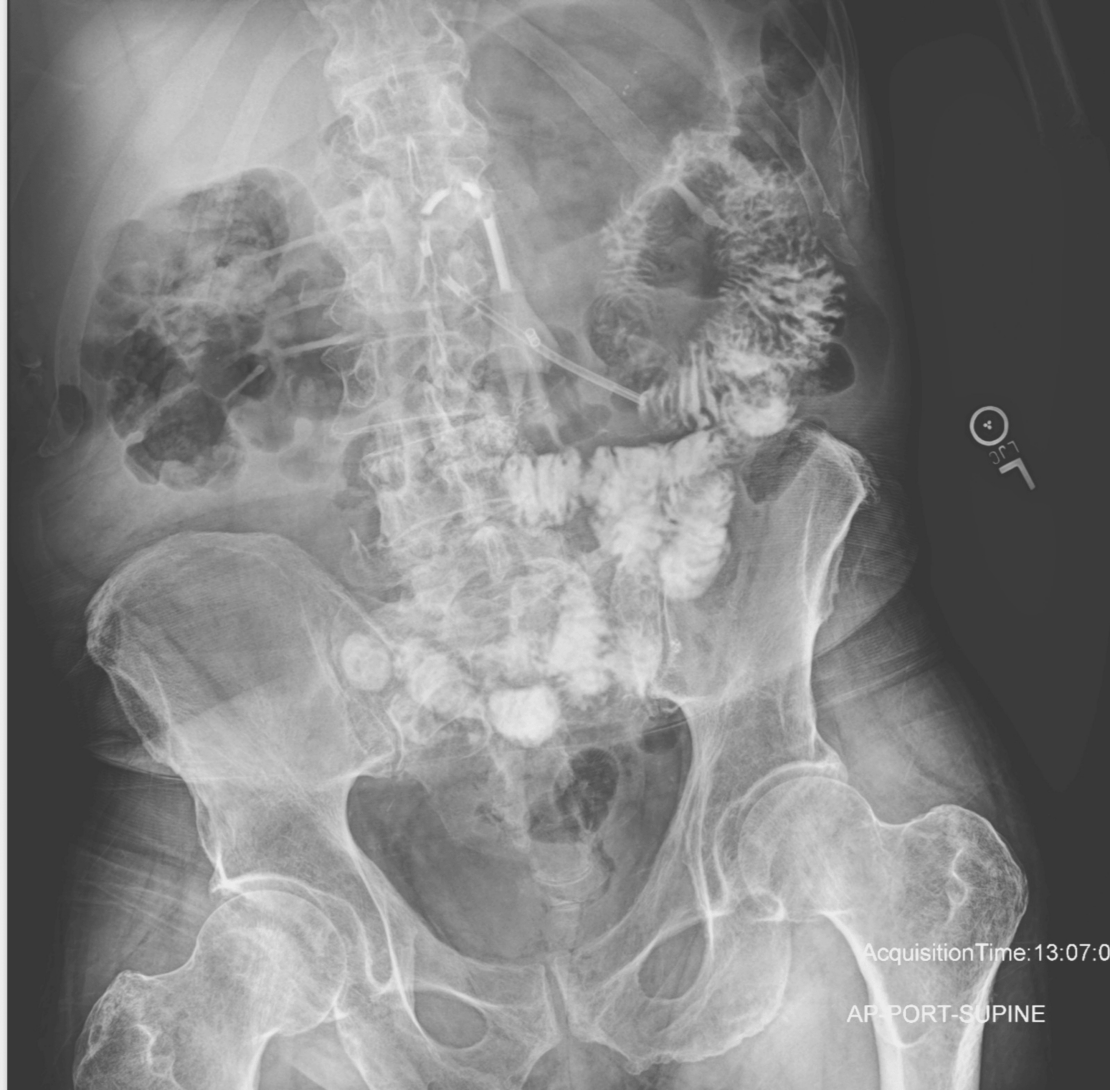
Abdomen and pelvis anterior-posterior single portable X-ray view Single portable AP, passive view of the abdomen and pelvis obtained after injection of contrast through the feeding tube, showing contrast within the PEG tube and multiple non-dilated small bowel loops over the left upper quadrant and mid-abdomen. No contrast was seen in the stomach or distal small bowel, and no extra luminal contrast was identified. PEG: Percutaneous endoscopic gastrostomy

The G-tube series, performed to assess tube placement, showed opacification of non-dilated proximal small bowel loops following contrast via the PEG tube, with no extraluminal contrast seen. Lack of contrast within the stomach is likely related to tube advancement as reported by the patient (Figure 1).

**Figure 2:**
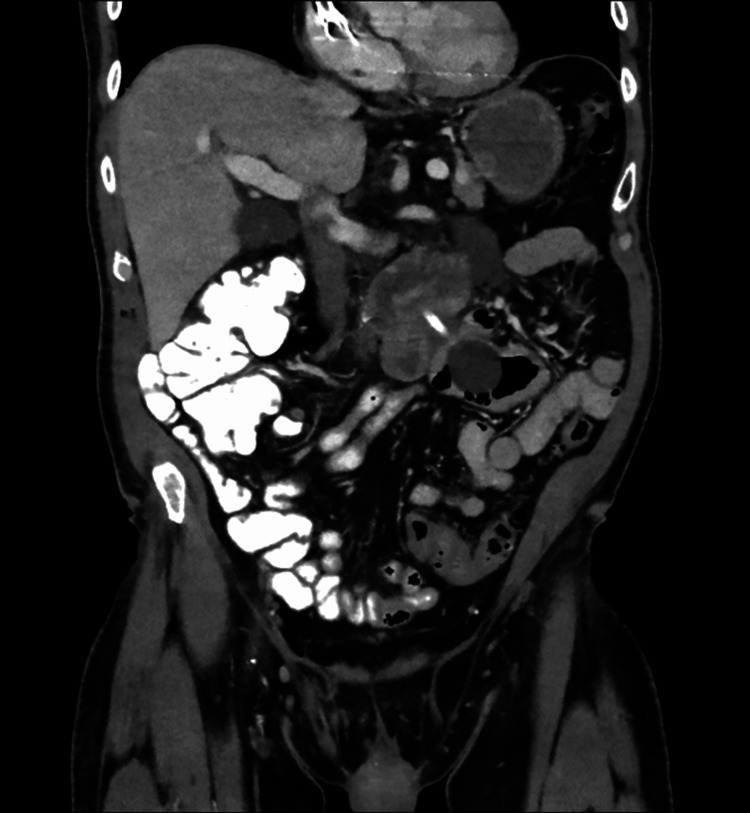
CT abdomen pelvis

**Figure 3:**
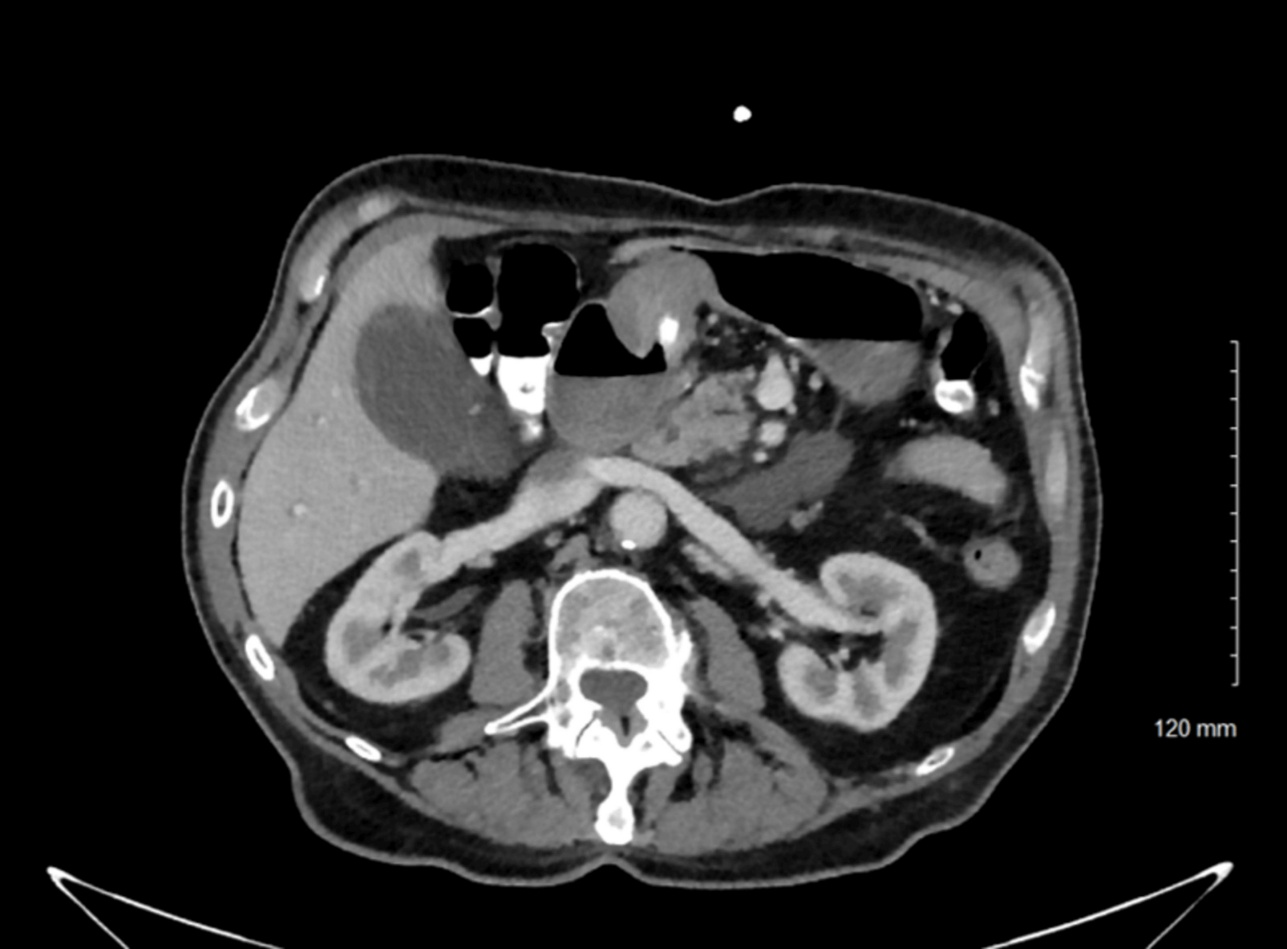
CT abdomen pelvis CT imaging showing pericholecystic and peripancreatic fluid concerning for acute panceatitis

A computed tomography (CT) scan of the abdomen and pelvis was done concerning acute pancreatitis, revealing pericholecystic and peripancreatic fluid but no evidence of gallbladder wall thickening or other findings suggestive of acute cholecystitis (Figures 2, 3). The combination of symptoms, elevated lipase, and imaging findings was consistent with acute pancreatitis, likely secondary to the obstructive process. We deduce that the PEG tube migration causes acute obstructive pancreatitis by compressing or obstructing the pancreatic duct, resulting in inflammation. 

## Discussion

PEG is commonly used as a mode of nutrition in patients who are unable to tolerate food by mouth and is a preferred method for long-term enteral nutrition. PEG tubes have been used for decades, and their insertion is safer when done under endoscopic guidance. However, studies have reported PEG tube placement-associated complications in about 4% to 25 % of patients [4,5]. Common complications reported are not limited to peristomal wound infection, granuloma formation, peritonitis, stoma leakage, aspiration pneumonia (due to esophageal reflux), hemorrhage, buried bumper syndrome, perforated viscus, necrotizing fasciitis, and colonic fistula (due to misplaced PEG tube); however, there have been few literature reports on the potential complication of pancreatitis in these patients [5].

Acute pancreatitis, a significant inflammatory condition of the pancreas, can be triggered by various etiologies, including gallstones, chronic alcohol use, and, less commonly, iatrogenic causes. Among the rarer iatrogenic causes is the migration of a PEG tube, as highlighted in this case. As with any surgical procedure, there can be complications. Retrograde migration of the gastrostomy tube into the abdominal wall or sinus tract is one such complication that has been described [5,6]. Another potential theory for PEG tube migration is from peristalsis leading to tube misplacement in the duodenum [4,7]. 

Buried bumper syndrome is a condition that can occur due to PEG tube migration, where the internal bumper of the PEG tube becomes buried in the gastric wall. This syndrome can lead to significant complications, including feeding intolerance, inflammation, and potentially perforation of the gastric wall if not identified early [8-10].

Acute pancreatitis in the context of this case is most likely induced by mechanical obstruction. The migration of the PEG tube may have caused compression or obstruction of the pancreatic duct. This obstruction can lead to the activation of pancreatic enzymes within the gland itself, initiating a cascade of events that culminates in inflammation and autodigestion of pancreatic tissue [1,6-8]. Such a mechanism is consistent with cases reported in the literature, where PEG tube migration into the duodenum or jejunum has similarly resulted in acute pancreatitis due to duodenal or pancreatic duct obstruction [4,9]. 

The rarity of this complication can be attributed to the design and placement of the PEG tube, which is intended to remain securely in the stomach. However, factors such as improper tube placement, excessive patient movement, different PEG tube bumper sizes based on device type and make, and significant weight changes can contribute to the tube migrating out of position. The current literature reports only a handful of cases where PEG tube migration resulted in acute pancreatitis, but these cases highlight the seriousness of the complication [4,8]. 

The diagnosis was confirmed through a combination of laboratory findings and imaging studies. Elevated lipase levels, which are highly sensitive and specific for acute pancreatitis, guided the diagnostic process. However, it was the imaging, particularly the CT scan, that provided definitive evidence of the PEG tube’s role in the pathology. The scan revealed peripancreatic fluid and signs of a likely obstructive process associated with the misplaced tube, which is consistent with other reported cases where imaging played a crucial role in identifying the mechanical obstruction caused by PEG tube migration [1,10-13]. 

The management of acute pancreatitis due to PEG tube migration is primarily focused on resolving the mechanical obstruction. In this case, deflation and repositioning of the PEG tube were sufficient to alleviate the obstruction, leading to a resolution of the patient’s symptoms and normalization of lipase levels. This outcome demonstrates that, when diagnosed early, the management of this complication can be straightforward and effective [13]. However, delayed diagnosis or intervention could result in more severe outcomes, including necrotizing pancreatitis, systemic inflammatory response syndrome, or even death [2,7]. 

This case emphasizes the importance of careful monitoring and maintenance of PEG tubes. Regular follow-up, including physical examination and imaging, when necessary, is crucial in detecting early signs of tube migration or other complications. Clinicians should be particularly vigilant in patients with risk factors for tube migration, such as those with significant weight loss or altered gastrointestinal anatomy [7]. Education of patients and caregivers on the signs of tube displacement and the potential complications is also essential in preventing severe outcomes [9]. 

## Conclusions

This case demonstrates the importance of recognizing PEG tube migration as a rare but significant cause of acute pancreatitis. Timely diagnosis through laboratory and imaging studies, followed by appropriate management to correct tube positioning, is essential in preventing complications and ensuring favorable outcomes. Regular monitoring of PEG tube position, patient education on potential complications, and careful follow-up are key to preventing similar events in the future. This case underscores the need for clinicians to consider PEG tube-related complications in the differential diagnosis of acute pancreatitis, particularly in vulnerable populations.
